# Sex differences in the development, treatment, and prognosis of multiple sclerosis in Switzerland

**DOI:** 10.3389/fnins.2026.1745599

**Published:** 2026-04-08

**Authors:** Timea-Chiara Annovazzi, Jens Kuhle, Pascal Benkert, Sabine Schaedelin, Joelle Schwarz, Enriqueta Vallejo-Yagüe, Carole Clair, Caroline Pot

**Affiliations:** 1 Health and Gender Unit, University Center for Primary Care and Public Health (Unisanté), University of Lausanne, Lausanne, Switzerland; 2 Multiple Sclerosis Centre and Research Center for Clinical Neuroimmunology and Neuroscience (RC2NB), Departments of Neurology, Biomedicine and Clinical Research, University Hospital and University of Basel, Basel, Switzerland; 3 Department of Clinical Research, University Hospital Basel, University of Basel, Basel, Switzerland; 4 Epidemiology, Biostatistics and Prevention Institute, University of Zurich, Zurich, Switzerland; 5 Institute of Primary Health Care (BIHAM), University of Bern, Bern, Switzerland; 6 Service of Neurology, Lausanne University Hospital and University of Lausanne, Lausanne, Switzerland

**Keywords:** body mass index, cross-sectional study, disability, disease progression, Expanded Disability Status Scale (EDSS), gender differences, multiple sclerosis, neurodegeneration

## Abstract

**Introduction:**

There has been growing recognition of potential differences in disease course and presentation between men and women with MS. This study examined sex differences in MS using data collected at study entry in the Swiss Multiple Sclerosis Cohort (SMSC).

**Methods:**

A cross-sectional analysis of the data from 1541 SMSC participants (June 2012–February 2022) with persons with relapsing-remitting MS or Clinically Isolated Syndrome (named relapsing type) and progressive MS including persons with Primary Progressive Multiple Sclerosis (PPMS) and Secondary Progressive Multiple Sclerosis (SPMS) was performed. Sociodemographic and clinical characteristics, disease history, and severity indicators were examined, focusing on sex differences within progressive and relapsing MS types, and comparing these MS types. Statistical analyses included Mann-Whitney U tests and chi-squared tests for group comparisons. Multivariate linear regression models were constructed to examine the independent association of sex with Expanded Disability Status Scale (EDSS) scores, adjusting for age, disease duration, treatment category, recent relapse, and body mass index (BMI).

**Results:**

Women represented 65.8% of the cohort (1,014/1,541). BMI was significantly lower in women than in men in the relapsing type and SPMS (relapsing: *p* < 0.001; SPMS: *p* = 0.001; PPMS: *p* = 0.86). Age at first symptoms differed by sex depending on MS type: women were younger in the relapsing group (29.7 vs. 31.4 years, *p* = 0.036), while men were younger in PPMS (42.3 vs. 47.7 years, *p* < 0.001), with no difference in SPMS (*p* = 0.5). In univariate analysis, men showed a trend toward higher disability levels at study entry in the relapsing type (*p* = 0.058), but no significant sex differences in EDSS were observed in progressive forms. In multivariate analysis, female sex showed a trend toward lower EDSS scores in relapsing MS after adjusting for clinical factors (β = −0.13, 95% CI: −0.26 to 0.005, *p* = 0.059) but was not associated with EDSS in PPMS (β = −0.09, *p* = 0.802) or SPMS (β = + 0.09, *p* = 0.816).

**Conclusion:**

This study identified sex differences in disease distribution, BMI and EDSS at their entry in the SMSC. These findings underscore the complexity of sex differences in MS and highlight the importance of prospective longitudinal studies with standardized severity assessments to clarify sex-specific disease trajectories and inform personalized treatment strategies.

## Introduction

Multiple sclerosis (MS) is a chronic immune-mediated disease that affects the central nervous system, causing a wide range of symptoms ranging from motor and sensory impairments to cognitive dysfunction (Compston and Coles, 2008). Other common symptoms include fatigue, visual, bowel and bladder symptoms (Compston and Coles, 2008). It is the most common neurological pathology affecting young adults according to the Multiple Sclerosis International Federation (Multiple Sclerosis International Federation [MSIF], 2013). An estimated 18,000 persons are currently living with this disease in Switzerland (Blozik et al., 2017; Iaquinto et al., 2024).

Multiple sclerosis affects women more than men, with women being two to three times more likely to develop MS (Dunn et al., 2015). However, this sex disparity reverses when considering disease severity: male has been associated with a higher risk of faster disease progression and disability accumulation (Ysrraelit and Correale, 2019). While the mechanisms underlying these sex-based differences remain incompletely understood, several hypotheses have been proposed (Harbo et al., 2013). Hormonal factors could play a role, with estrogen demonstrating neuroprotective and immunomodulatory effects that may influence both MS development and progression (Gold and Voskuhl, 2009; Bove and Chitnis, 2013). In line with this hormonal hypothesis, pregnancy-related sex hormones increases have been associated with MS symptom improvement (Gold and Voskuhl, 2009). Genetic factors also appear to contribute to sex-based differences in MS susceptibility. Certain genetic variants linked to MS are more commonly associated in women than in men (Voskuhl and Gold, 2012; International Multiple Sclerosis Genetics Consortium, 2019) suggesting sex-specific genetic risk profiles that may interact with hormonal and factors to influence disease development. Environmental factors have also been implicated in the higher prevalence of MS in women (Klein and Burton, 2014). The risk of developing MS increases after exposure to the Epstein-Barr-Virus (EBV) even more when the infection is symptomatic (Handel et al., 2010). Women may be more susceptible to EBV than men (Ascherio and Munger, 2010; Correale et al., 2013). Other social and lifestyle factors, such as stress (Mohr and Pelletier, 2006), use of oral contraception (Leray et al., 2016), smoking (O’Gorman and Broadley, 2016) and diet (Ghadirian et al., 1998), also may play a role in the development and progression of MS. Smoking, in particular, has been associated with more rapid disease progression in MS (O’Gorman and Broadley, 2016), with differential prevalence observed between men and women (Office Fédéral de la Statistique [OFS], 2025).

The earlier disease severity observed in men appears to reflect distinct pathophysiological mechanisms. Male sex has been associated with higher rates of neurodegeneration and axonal damage (Magyari and Koch-Henriksen, 2022) with shorter time to conversion to secondary progressive MS in untreated patients (Alvarez-Sanchez and Dunn, 2023). Whereas female sex is linked with more pronounced inflammatory activity (Magyari and Koch-Henriksen, 2022), a higher relapse rate and gadolinium-enhancing lesions (Nesbitt et al., 2025). In terms of cognitive decline men exhibit greater cognitive impairment and more severe neurocognitive deficits than women (Alvarez-Sanchez and Dunn, 2023). However, the impact of menopause on disability progression remains unclear (Bridge et al., 2025; Silverman et al., 2025). Notably, these sex differences appear to become less prominent in late-onset MS (age > 50 years) during the perimenopausal or postmenopausal period (Nesbitt et al., 2025). The decline in estrogen levels following menopause may suppress neuroprotective pathways and seems to shift the disease course in women toward more degenerative processes, potentially exacerbating symptoms (Nesbitt et al., 2025). Consequently, the distinction between the inflammatory female phenotype and the neurodegenerative male phenotype may diminish with age, though differentiating menopausal effects from aging effects remains challenging (Nesbitt et al., 2025).

This study aims to evaluate the population of the Swiss Multiple Sclerosis Cohort (SMSC) at cohort entry, particularly comparing disease severity in men and women, grouped by disease type.

## Materials and methods

### Study design and data source

This cross-sectional study uses entry data from the ongoing prospective Swiss Multiple Sclerosis Cohort (SMSC), a multicenter cohort study conducted across eight Swiss centers, including the Cantonal Hospital of Aarau, the University Hospitals of Basel, Berne, Geneva, Lausanne and Zürich, the Regional Hospital of Lugano, and the Cantonal Hospital of St. Gallen. A detailed protocol has been described in a previous publication (Disanto et al., 2016). The recruitment of this observational cohort started in June 2012 (Disanto et al., 2016). Participants of the SMSC provided informed consent and are diagnosed with Relapsing-Remitting Multiple Sclerosis (RRMS), Secondary Progressive Multiple Sclerosis (SPMS) or Primary Progressive Multiple Sclerosis (PPMS) [according to the 2010 revised McDonald criteria (Disanto et al., 2016)], Clinically Isolated Syndrome (CIS), neuromyelitis optica or radiologically isolated syndrome. SMSC participants’ inclusion criteria also encompass being untreated, treated, starting or switching to a disease modifying treatment (DMT) and willingness to attend hospital visits every 6 or 12 months. In the SMSC, demographic, clinical and cerebrospinal fluid data is collected prospectively following a pre-established protocol previously described (Disanto et al., 2016). At study entry, demographics, family MS history, pregnancy history, neurostatus including the Expanded Disability Status Scale (EDSS), laboratory tests, pre-study entry relapses, medical events, and treatment information were collected (Disanto et al., 2016). Neurofilament Light Chain (NfL) levels in serum and cerebrospinal fluid (CSF) are available as described previously (Benkert et al., 2022). Data at SMSC entry represents a cross-sectional assessment and were not linked to a specific time from diagnosis.

Data access was obtained through institutional agreement with the SMSC committee. This study was conducted with approval from the Ethics Committee of each participating hospital (NCT02433028). All participants provided written informed consent (Disanto et al., 2016).

### Study population

For this study, inclusion required participants to be diagnosed with either CIS, RRMS, SPMS or PPMS. Patients diagnosed with neuromyelitis optica or radiologically isolated syndrome were excluded from the analysis, (Wingerchuk et al., 2015; Barboza et al., 2021).

Participants were stratified by disease type into three categories: relapsing MS (RRMS and CIS), PPMS, and SPMS. Given the differences in clinical trajectories of PPMS and SPMS, they were analyzed separately rather than as a combined progressive group (Fox et al., 2017).

Additionally, within each disease type, participants were grouped by administrative sex (men, women). Self-reported administrative sex was collected as a dichotomous variable. We recognize that both biological sex and social gender may contribute to the observed differences. As our data lacked detailed measures of gender identity and other gender or biological dimensions, we relied on the binary administrative categories, acknowledging that the findings may reflect effects of biological sex, social gender, or their interaction.

### Study variables

Study variables were organized into three categories:

1. Population description: age at study entry, body mass index (BMI), ethnicity (Caucasian/non-Caucasian), MS family history (yes/no/unknown), and disease duration at study entry.
2. Disease history: age at first symptoms, time between first symptoms and diagnosis, number of relapses in the 2 years prior to study entry and treatment at study entry. Relapses were defined according to standard clinical criteria as clinician-confirmed episodes of new or worsening neurological symptoms lasting at least 24 h, in the absence of fever or infection, and consistent with an acute inflammatory demyelinating event. All relapses were documented by treating neurologists during clinical assessments. For descriptive purposes, we calculated the median number of relapses occurring in the 2 years prior to study entry for each patient. For regression analyses, we created a binary variable indicating recent inflammatory activity, defined as the occurrence of at least one relapse within 3 months prior to study entry (yes/no). Treatment at study entry was categorized as currently no treatment, sphingosine-1-phosphate receptor modulators (fingolimod), monoclonal antibodies, beta interferons, dimethyl fumarate (tecfidera), glatiramer acetate (copaxone), teriflunomide (aubagio), cladribine (mavenclad), mitoxantrone (novantron), glucocorticoids (Solumedrol), or azathioprine (imurek). For regression analyses, treatments were recategorized into efficacy-based classes (1) Untreated (no disease-modifying therapy); (2) Platform compounds (interferon beta preparations and glatiramer acetate); (3) Oral therapies (fingolimod, dimethyl fumarate, teriflunomide, cladribine); (4) High-efficiency monoclonal antibody therapies (alemtuzumab, natalizumab, ocrelizumab, rituximab); and (5) Other (mitoxantrone, azathioprine, glucocorticoids, participation in randomized clinical trials, other immunosuppressants). This categorization was implemented to reduce the number of treatment variables in multivariable models, improve statistical power, and align with current classification systems used in a previous paper published using SMSC data (Benkert et al., 2022).
3. Disease severity indicators (study outcomes): EDSS score at study entry (0–3.5, 4–6.5, >6.5) served as the primary outcome measure. Standardized clinical assessments with functional system score and EDSS score calculations were performed by certified raters.^1^ For descriptive purposes, three groups were defined based on EDSS: 0–3.5 (mild disability), 4.0–6.5 (moderate disability, limited walking ability), and >6.5 (severe disability, wheelchair required or worse) (Bastos et al., 2024) and presented as mean ± standard deviation. For regression analyses, EDSS was analyzed as a continuous variable (linear regression) to maximize statistical power. NfL is a neuroaxonal cytoskeletal protein released into cerebrospinal fluid and blood following neuronal damage (Disanto et al., 2016). Hence serving as a biomarker of neuroaxonal injury, disease activity, and treatment response in MS. NfL Z scores express the deviation of the (age and BMI) adjusted sNfL from values in the healthy control’s population in terms of number of standard deviations from the mean (Benkert et al., 2022). Due to limited sample size, particularly among patients with recent relapse activity, NfL analyses are presented as exploratory findings in Supplementary materials and should therefore be interpreted with caution (Supplementary Tables 1–3).

### Exposure

Comparison between women and men was done within each MS type group.

### Statistical analysis

Participants were first categorized by MS type. Overall and within each MS type, the study population was described, including sociodemographic, clinical characteristics, disease history, along with the severity indicators (the outcomes of interest). Overall participant characteristics are presented in Supplementary Tables 4–7, including ethnicity distribution by sex, disease duration at study entry by sex, sex distribution by MS type, and sex distribution by MS disease course.

Continuous variables were presented as medians with IQR. Categorical variables were presented as frequencies and proportions. The number of relapses in the 2 years prior to baseline showed a highly skewed distribution with a large proportion of zero values (Relapsing: 41%; Progressive: 87.9%). Given the non-normal distribution (Shapiro-Wilk test *p* < 0.001), we reported the median with interquartile ranges for this variable. Additionally, to better visualize the distribution, we categorized the number of relapses into five groups: 0, 1, 2, 3, and ≥4 relapses, presented as frequencies and proportions.

Mann-Whitney U tests were used to compare continuous variables between men and women within each MS type. Chi-squared tests were applied for categorical variables, including comparisons of sex distribution across MS types and ethnicity distribution between sexes. A *p*-value < 0.05 was considered statistically significant.

To examine sex-related differences in disability while controlling for potential confounders, we constructed linear regression models with EDSS (range 0–10) as the continuous outcome variable and female sex as the predictor of interest. We specified three sequential models for each MS type to assess the impact of progressive adjustment for confounders. The unadjusted model included female sex as the sole predictor. Model A (age-adjusted) adjusted for age at study entry only. Model B (fully adjusted) examined the association between female sex and EDSS while adjusting for age at study entry, disease duration at study entry, treatment efficacy category (5-level categorical variable: currently no treatment, platform compounds, oral therapies, high-efficiency monoclonal antibodies, other), recent inflammatory activity (relapse within 3 months prior to study entry), and body mass index (BMI). Missing data was handled using available case analysis (pairwise deletion), with statistical tests performed on non-missing observations for each variable. The amount of missing data is reported for each variable. Most clinical variables showed low proportions of missing data (<5%), with the exception of BMI, which exhibited a moderate level of missingness (approximately 9% in relapsing MS and 17%–18% in progressive MS), and serum NfL measurements, particularly those obtained within 3 months of a relapse, which were missing in the majority of participants. For the small proportion of missing data in variables other than BMI (<3%), listwise deletion was applied. To assess potential differences between participants with and without available data, baseline characteristics were compared using appropriate statistical tests (Supplementary Tables 8, 9). Missingness in BMI was not significantly associated with age, sex, EDSS, disease duration, or treatment category. In contrast, missingness in NfL variables was significantly associated with MS type and disease duration, reflecting differences in clinical indications and availability of biological sampling rather than random missingness. Given the proportion of missing BMI data and the absence of strong associations with key clinical variables, BMI was imputed for the multivariate analyses with its median value of (24.8 kg/m^2^) for missing values. For further information, the Supplementary Tables 8, 9 includes a table reporting the percentage of missing data by sex and MS subtype; and a table comparing baseline characteristics (age, sex, and MS subtype) between participants with and without missing data.

All statistical analyses were conducted using RStudio 2024.04.1 (V4.4.2, Boston, MA) (R Foundation for Statistical Computing, 2022).

## Results

**FIGURE 1:**
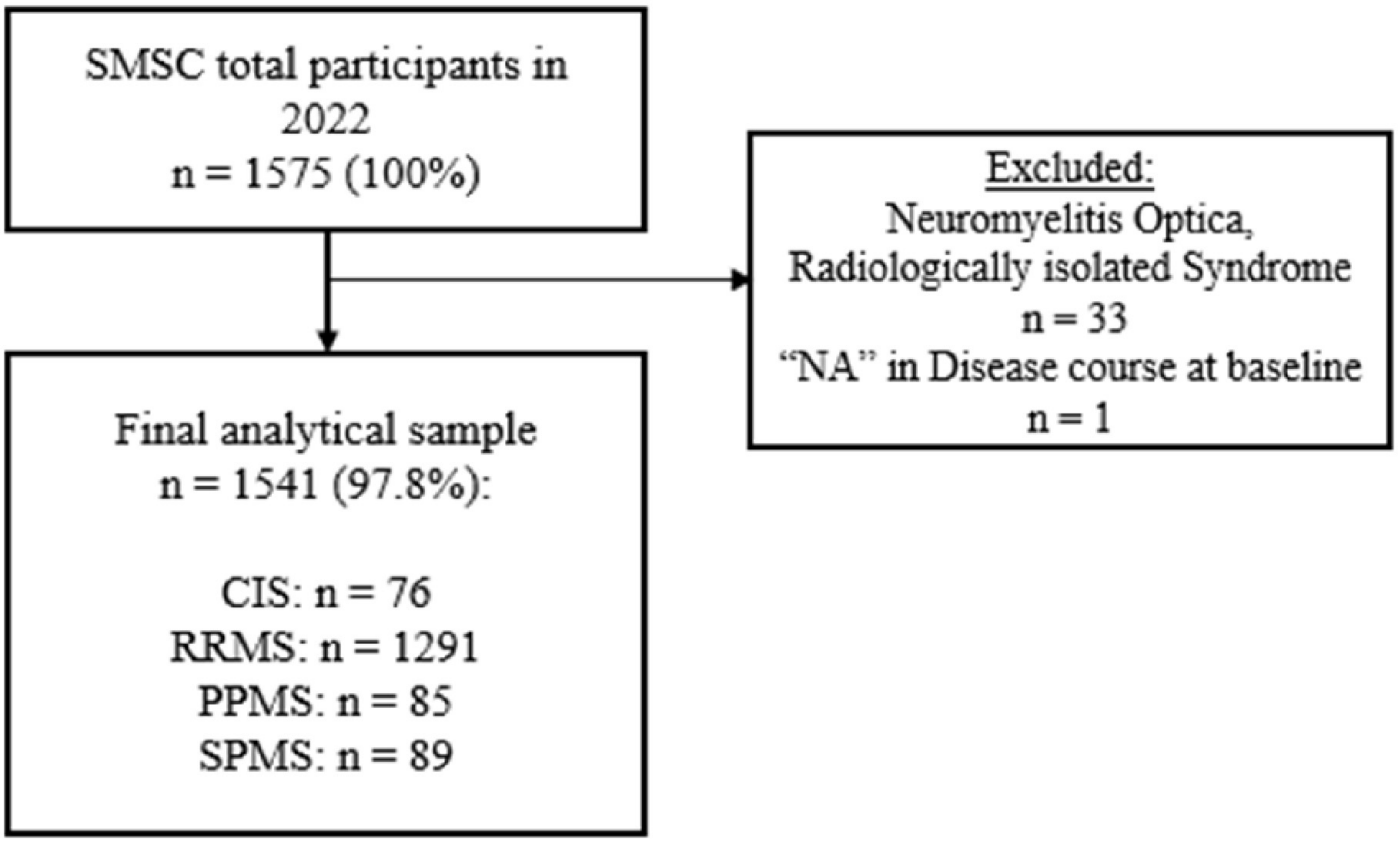
Study flowchart depicting the inclusion of participants in this study.

A total of 1,575 patients were enrolled in the prospective SMSC cohort until February 2022 (Figure 1). After excluding 34 patients (2.2%) who did not meet the inclusion criteria, the final study sample included 1,541 participants with 76 having CIS, 1291 having RRMS, 85 having PPMS and 89 having SPMS (Figure 1).

Sex distribution differed significantly across MS types (*p* < 0.001), with women representing 67.8% of the relapsing MS group (927 out of 1,367) and 50.0% of the progressive MS group (87 out of 174). Sex distributions within each MS disease course at study entry are shown in Supplementary Table 4. Disease duration at study entry was similar between men and women (men: median 6.8 years [IQR: 1.7–14.1], *n* = 521; women: median 6.9 years [IQR: 2.1–14.0], *n* = 1,009; *p* = 0.8). Ethnicity distribution was similar between men and women (*p* = 0.4), with most participants being Caucasian (men: 98.7%; women: 97.9%) (see Supplementary Tables 1–3).

### Relapsing MS characteristics

In the relapsing type, median BMI at study entry was lower in women than in men (23.6 vs. 25.2 kg/m^2^, *p* < 0.001) (Table 1). Median age at study entry, ethnicity, MS family history, and median disease duration at study entry were comparable between men and women. Median age at first symptoms was slightly lower in women than in men (29.7 vs. 31.4 years, *p* = 0.036). Median time between first symptoms and diagnosis was similar between sexes. The distribution of relapses in the 2 years prior to study entry showed no significant sex differences (*p* = 0.8), with approximately 41% reporting no relapses and 50% reporting four or more relapses. Treatment patterns at study entry were comparable between men and women (*p* = 0.2). The most common treatments were Sphingosine-1-phosphate receptor modulators (Gilenya) (28.7%), followed by monoclonal antibodies (24.5%) and no current treatment (26.9%).

**TABLE 1:** Relapsing MS type – population description and study outcomes.

Relapsing MS (RRMS/CIS)	*n*	Men	Women	*P*-value	Overall
		*N* = 440	*N* = 927		*N* = 1,367
Population description:					
Median age at study entry, years (IQR)	1,367	39.5 (32.0, 47.8)	39.1 (30.5, 48.1)	0.5	39.3 (31.1, 47.9)
Median BMI, kg/m^2^, (IQR)	1,245	25.2 (22.8, 28.5)	23.6 (21.2, 26.7)	<0.001	24.1 (21.5, 27.3)
Missing, *n* %	122 (8.9)	40 (9.1)	82 (8.8)		
Ethnicity, *n* (%)	1,367			0.5	1,339 (98)
Caucasian		433 (98)	906 (98)		
Non-Caucasian		7 (1.6)	21 (2.3)		28 (2.0)
MS family history *n* (%)	1,367			0.11	
	No	323 (73)	728 (79)		1,051 (77)
	Yes	67 (15)	113 (12)		180 (13)
	Unknown	50 (11)	86 (9.3)		136 (9.9)
Median disease duration at study entry, years (ICR)	1,359	5.8 (1.2, 12.4)	6.2 (1.8, 12.8)	0.3	6.0 (1.6, 12.7)
Missing, *n* %	8 (0.8)	4 (0.9)	4 (0.4)		
Disease history:					
Median age at first symptoms, years (IQR)	1,362	31.4 (25.1, 37.8)	29.7 (23.7, 37.6)	0.036 0.7	30.4 (24.1, 37.7) 0.7 (0.1, 2.8)
Missing, *n* %	5 (0.4)	3 (0.7)	2 (0.2)		
Median time between first symptoms and diagnosis, years (IQR)	1,320	0.7 (0.1, 3.0)	0.8 (0.1, 2.7)		
Missing, *n* %	47 (3.4)	22 (5.0)	25 (2.7)	0.8	3.0 (0.0, 9.0)
Median number of relapses 2 years prior to study entry, (IQR)	1,367	4.0 (0.0, 9.0)	3.0 (0.0, 10.0)		
Number of relapses (categories) 2 years prior to study entry, *n* %	1,367			0.5	
0		180 (41.0%)	385 (42.0%)		565 (41.0%)
1		12 (2.7%)	32 (3.5%)		44 (3.2%)
2		15 (3.4%)	39 (4.2%)		54 (4.0%)
3		6 (1.4%)	19 (2.0%)		25 (1.8%)
≥4		227 (52.0%)	452 (49.0%)		679 (50.0%)
Treatment at study entry*, *n* %	1,367			0.2	
Currently no treatment		124 (28.2)	244 (26.3)		368 (26.9)
Sphingosine-1-phosphate receptor modulator (Gilenya)		128 (29.1)	264 (28.5)		392 (28.7)
Monoclonal antibodies		99 (22.5)	236 (25.5)		335 (24.5)
Beta interferons		40 (9.1)	77 (8.3)		117 (8.6)
Dimethyl fumarate		27 (6.1)	51 (5.5)		78 (5.7)
Glatiramer acetate		8 (1.8)	39 (4.2)		47 (3.4)
Teriflunomide		9 (2.0)	8 (0.9)		17 (1.2)
Purine analogue (cladribine)		3 (0.7)	4 (0.4)		7 (0.5)
Mitoxantron		1 (0.2)	0 (0.0)		1 (0.1)
Glucocorticoid (Solumedrol)		1 (0.2)	1 (0.1)		2 (0.1)
Azathioprine		0 (0.0)	3 (0.3)		3 (0.2)
Study outcome, disease severity indicators:					
EDSS at study entry, *n* %	1,360	377 (85.9)	816 (88.6)	0.058	1,193 (87.7)
0–3.5					
4–6.5		60 (13.7)	105 (11.4)		165 (12.1)
>6.5		2 (0.5)	0 (0)		2 (0.1)
Missing, *n* %	7 (0.5)	1 (0.2)	6 (0.6)		
Mean EDSS at study entry, ±SD	1360	2.3 ± 1.4	2.2 ± 1.3	0.156	2.2 ± 1.3
Missing, *n* %	7 (0.5)	1 (0.2)	6 (0.6)		

MS, multiple sclerosis; BMI, body mass index; EDSS, Expanded Disability Status Scale; Statistics (medians, interquartile ranges, percentages) and *p*-values were calculated using available data for each variable, excluding missing values. *Each participant has only one treatment at study entry.

Expanded Disability Status Scale scores at study entry showed a trend toward higher level in men (*p* = 0.058). Most women (88.6%) and men (85.9%) had study entry EDSS scores of 0–3.5, while 11.4% of women and 13.7% of men scored 4–6.5, and only 0.5% of men and 0.2% women scored > 6.5.

### PPMS characteristics

The PPMS subgroup comprised 85 patients (49 men, 36 women) (Table 2). Median age at study entry was 52.3 years (IQR 45.3–62.3) in men and 57.3 years (IQR 51.6–61.3) in women (*p* = 0.24). Median BMI was 24.0 kg/m^2^ (IQR 21.9–26.8) in men and 23.5 kg/m^2^ (IQR 20.4–27.4) in women (*p* = 0.86). BMI data were missing for 18% of patients. MS family history was negative in 64 patients (75.3%), positive in 12 (14.1%), and unknown in 9 (10.6%) (*p* = 0.3 between sexes). Median disease duration at study entry was 9.5 years (IQR 4.2–16.3) in men and 8.9 years (IQR 4.4–12.4) in women (*p* = 0.55). Median age at first symptoms was 42.3 years (IQR 32.1–48.1) in men and 47.7 years (IQR 38.9–53.5) in women (*p* < 0.001). Median time between first symptoms and diagnosis was 2.2 years (IQR 1.0–4.3) in men and 3.7 years (IQR 1.2–5.4) in women (*p* = 0.5). Median number of relapses in the 2 years prior to baseline was 0 (IQR 0–0) for both sexes (*p* = 0.4). No relapses were reported by 78 patients (91.8%), while 6 (7.1%) reported four or more relapses (*p* = 0.2 between sexes). At study entry, 55 patients (64.7%) were untreated. Among treated patients, 16 (18.8%) received ocrelizumab, 7 (8.2%) rituximab, 2 (2.4%) cladribine, 1 (1.2%) each received teriflunomide, fingolimod, interferon beta, Solumedrol, and dimethyl fumarate (*p* = 0.5 between sexes).

**TABLE 2:** Primary Progressive Multiple Sclerosis (PPMS) type – population description and study outcomes.

PPMS	*n*	Men (*N* = 49)	Women (*N* = 36)	*P*-value	Overall (*N* = 85)
Population description:					
Median age at study entry, years (IQR)	85	52.3 (45.3, 62.3)	57.3 (51.6, 61.3)	0.24	56.4 (48.1, 62.3)
Median BMI, kg/m^2^ (IQR)	70	24 (21.9, 26.8)	23.5 (20.4, 27.4)	0.86	24 (21.2, 26.8)
Missing, *n* %	15 (18)	10 (20.4)	5 (13.9)		
Ethnicity, *n* %	85			NA	
Caucasian		49 (100)	36 (100)		0 (100)
Non-Caucasian		0 (0)	0 (0)		0 (0)
MS family history, *n* %	85			0.3	
No		39 (79.6)	25 (69.4)		64 (75.3)
Yes		7 (14.3)	5 (13.9)		12 (14.1)
Unknown		3 (6.1)	6 (16.7)		9 (10.6)
Median disease duration at study entry, years (IQR)	83	9.5 (4.2, 16.3)	8.9 (4.4, 12.4)	0.6	8.9 (4.2, 15.3)
Missing, *n* %	2 (2.4)	1 (2)	1 (2.8)		
Disease history:					
Median age at first symptoms, years (IQR)	84	42.3 (32.1, 48.1)	47.7 (38.9, 53.5)	>0.001	44.1 (35.6, 51.5)
Missing, *n* %		0 (0)	1 (2.8)		1 (1.2)
Median time between first symptoms and diagnosis, years (IQR)	83	2.2 (1, 4.3)	3.7 (1.2, 5.4)	0.5	2.7 (1, 5.1)
Missing, *n* %	2 (2.4)	1 (2)	1 (2.8)		
Median number of relapses 2 years prior to baseline, (IQR)	85	0 (0, 0)	0 (0, 0)	0.4	0 (0, 0)
Number of relapses (categories), *n* %	85			0.2	
0		44 (89.8)	34 (94.4)		78 (91.8)
1		0 (0)	0 (0)		0 (0)
2		0 (0)	0 (0)		0 (0)
3		0 (0)	1 (2.8)		1 (1.2)
≥4		5 (10.2)	1 (2.8)		6 (7.1)
Treatment at study entry*, 2 years prior to baseline, *n* %	85			0.5T	
Currently no treatment		33 (67.3)	22 (61.1)		55 (64.7)
Rituximab		3 (6.1)	4 (11.1)		7 (8.2)
Ocrelizumab		10 (20.4)	6 (16.7)		16 (18.8)
Cladribine		1 (2)	1 (2.8)		2 (2.4)
Teriflunomide		1 (2)	0 (0)		1 (1.2)
Fingolimod		1 (2)	0 (0)		1 (1.2)
Interferon B		0 (0)	1 (2.8)		1 (1.2)
Solumedrol		0 (0)	1 (2.8)		1 (1.2)
Dimethyl fumarate		0 (0)	1 (2.8)		1 (1.2)
Study outcome, disease severity indicators:					
EDSS at study entry, *n* %	85	12 (24.5)	8 (22.2)	0.2	
0–3.5					20 (23.5)
4–6.5		30 (61.2)	27 (75)		57 (67.1)
>6.5		7 (14.3)	1 (2.8)		8 (9.4)
Missing, *n* %	0 (0)	0 (0)	0 (0)		
Mean EDSS at study entry, ±SD	85	4.9 ± 1.6	4.8 ± 1.4	0.739	4.8 ± 1.5
Missing, *n* %	0 (0)	0 (0)	0 (0)		

MS, multiple sclerosis; BMI, body mass index; EDSS, Expanded Disability Status Scale; Statistics (medians, interquartile ranges, percentages) and *p*-values were calculated using available data for each variable, excluding missing values. *Each participant has only one treatment at study entry.

Expanded Disability Status Scale at study entry was 0–3.5 in 20 patients (23.5%), 4.0–6.5 in 57 (67.1%), and >6.5 in 8 (9.4%) (*p* = 0.2 between sexes).

### SPMS characteristics

The SPMS subgroup comprised 89 patients (38 men, 51 women) (Table 3). Median age at study entry was 54.6 years (IQR 50.0–64.5) in men and 56.6 years (IQR 50.2–66.9) in women (*p* = 0.61). Median BMI was 26.0 kg/m^2^ (IQR 23.6–28.6) in men and 22.2 kg/m^2^ (IQR 20.7–25.0) in women (*p* = 0.001). BMI data was missing for 18% of patients. MS family history was negative in 63 patients (70.8%), positive in 11 (12.4%), and unknown in 15 (16.9%) (*p* = 0.7 between sexes). Median disease duration at study entry was 19.9 years (IQR 13.2–30.3) in men and 27.2 years (IQR 19.3–33.7) in women (*p* = 0.068). Median age at first symptoms was 31.8 years (IQR 22.9–43.7) in men and 28.9 years (IQR 24.1–37.2) in women (*p* = 0.5). Median time between first symptoms and diagnosis was 1.8 years (IQR 0.4–7.5) in men and 1.6 years (IQR 0.1–4.4) in women (*p* = 0.2). Median number of relapses in the 2 years prior to baseline was 0 (IQR 0–0) for both sexes (*p* = 0.3). No relapses were reported by 75 patients (84.3%), while 13 (14.6%) reported four or more relapses (*p* = 0.4 between sexes). At study entry, 44 patients (49.4%) were untreated. The most common treatments among the 45 treated patients were interferon beta (*n* = 11, 12.4%), followed by mitoxantrone, ocrelizumab, and rituximab (*n* = 7 each, 7.9%), glatiramer acetate and fingolimod (*n* = 3 each, 3.4%), cladribine, methylprednisolone, and dimethyl fumarate (*n* = 2 each, 2.2%), and azathioprine (*n* = 1, 1.1%) (*p* = 0.1 between sexes).

**TABLE 3:** Secondary Progressive Multiple Sclerosis (SPMS) type – population description and study outcomes.

SPMS	*n*	Men (*N* = 38)	Women (*N* = 51)	*P*-value	Overall (*N* = 89)
Population description					
Median age at study entry years (IQR)	89	54.6 (50–64.5)	56.6 (50.2–66.9)	0.61	56 (49.5–66.3)
Median BMI kg/m^2^ (IQR)	73	26 (23.6–28.6)	22.2 (20.7–25)	0.001	24.2 (21.2–27.2)
Missing, *n* (%)	16	5 (13.2)	11 (21.6)		
Ethnicity *n* (%)	89			NA	
Caucasian		38 (100)	51 (100)		89 (100)
Non-Caucasian		0 (0)	0 (0)		0 (0)
MS family history *n* (%)	89			0.7	
No		26 (68.4)	37 (72.5)		63 (70.8)
Yes		6 (15.8)	5 (9.8)		11 (12.4)
Unknown		6 (15.8)	9 (17.6)		15 (16.9)
Median disease duration at study entry years (IQR)	88	19.9 (13.2–30.3)	27.2 (19.3–33.7)	0.068	24.9 (16.7–32.5)
Missing, *n* (%)	1	1 (2.6)	0 (0)		
Disease history					
Median age at first symptoms years (IQR)	88	31.8 (22.9–43.7)	28.9 (24.1–37.2)	0.5	30.4 (23.7–40.5)
Missing, *n* (%)	1 (2.6)	0 (0)		1 (1.1)
Median time between first symptoms and diagnosis years	88	1.8 (0.4–7.5)	1.6 (0.1–4.4)	0.2	1.8 (0.3–5.1)
Missing, *n* (%)	1	1 (2.6)	0 (0)		
Mean number of relapses 2 years prior to baseline	89	0 (0–0)	0 (0–0)	0.3	0 (0–0)
Number of relapses (categories) 2 years prior to baseline, *n* (%)	89			0.4	
0		34 (89.5)	41 (80.4)		75 (84.3)
1		0 (0)	0 (0)		0 (0)
2		0 (0)	0 (0)		0 (0)
3		0 (0)	1 (2)		1 (1.1)
≥4		4 (10.5)	9 (17.6)		13 (14.6)
Treatment at study entry* *n* (%)	89			0,1	
Currently no treatment		19 (50)	25 (49)		44 (49.4)
Rituximab		2 (5.3)	5 (9.8)		7 (7.9)
Ocrelizumab		1 (2.6)	6 (11.8)		7 (7.9)
Glatiramer acetate		1 (2.6)	2 (3.9)		3 (3.4)
Fingolimod		1 (2.6)	2 (3.9)		3 (3.4)
Azathiopine		0 (0)	1 (2)		1 (1.1)
Betaferon		6 (15.8)	2 (3.9)		8 (9)
Caldribin		1 (2.6)	1 (2)		2 (2.2)
Mitoxantrone		6 (15.8)	1 (2)		7 (7.9)
Interferon		0 (0)	3 (5.9)		3 (3.4)
Solumedrol		0 (0)	2 (3.9)		2 (2.2)
Dmethyl fumarate		1 (2.6)	1 (2)		2 (2.2)
Study outcome disease severity indicators					
EDSS at study entry *n* (%)	88			0.8	
0–3.5		3 (7.9)	4 (7.8)		7 (7.9)
4–6.5	24 (63.2)	30 (58.8)	54 (60.7)	
>6.5	10 (26.3)	17 (33.3)	27 (30.3)	
Missing, *n* (%)	1	1 (2.6)			0 (0)
Mean EDSS at study entry, ±SD	88	5.8 ± 1.7	5.8 ± 1.4	0.893	5.8 ± 1.5
Missing, *n* (%)	1	1 (2.6)	0 (0)		

MS, multiple sclerosis; BMI, body mass index; EDSS, Expanded Disability Status Scale; Statistics (medians, interquartile ranges, percentages) and *p*-values were calculated using available data for each variable, excluding missing values. *Each participant has only one treatment at study entry.

### Multivariate analysis

Sequential adjustment models revealed MS type-specific patterns in the association between female sex and EDSS (Table 4).

**TABLE 4:** Association between female sex and EDSS scores: sequential adjustment models by MS type.

MS type	Model	Female sex β (95% CI)	*P*-value	*N*
Relapsing MS (*n* = 1352)	Unadjusted	−0.12 (−0.27; 0.03)	0.128	1352
	Age-adjusted (Model A)	−0.10 (−0.24; 0.05)	0.184	1352
	Fully adjusted (Model B)	−0.13 (−0.26; 0.005)	0.059	1352
PPMS (*n* = 83)	Unadjusted	−0.16 (−0.84; 0.52)	0.644	83
	Age-adjusted (Model A)	−0.19 (−0.88; 0.50)	0.580	83
	Fully adjusted (Model B)	−0.09 (−0.78; 0.61)	0.802	83
SPMS (*n* = 87)	Unadjusted	0.04 (−0.63; 0.70)	0.911	87
	Age-adjusted (Model A)	0.09 (−0.58; 0.76)	0.790	87
	Fully adjusted (Model B)	0.09 (−0.65; 0.82)	0.816	87

β, Regression coefficient for female sex vs. male (reference). Negative values indicate lower EDSS (less disability) in women. Unadjusted: Female sex only, Model A (Age-adjusted): Adjusted for age at study entry, Model B (Fully adjusted): Adjusted for age, disease duration, treatment efficacy category, recent inflammatory activity (relapse within 3 months), and BMI, PPMS, Primary Progressive MS; SPMS, Secondary Progressive MS.

In relapsing MS, while unadjusted and age-adjusted models showed no significant sex-related differences in EDSS (*p* = 0.128 and *p* = 0.184, respectively), full adjustment for confounders revealed a borderline trend toward lower EDSS in women (β = −0.130, 95% CI: −0.26 to 0.005, *p* = 0.059). In contrast, no association between female sex and EDSS was observed in PPMS or SPMS across any level of adjustment (all *p* > 0.58).

## Discussion

This cross-sectional study on data from the SMSC showed higher proportion of women in relapsing forms of MS compared to men. Women had significantly lower BMI in Relapsing MS and SPMS, and had their first symptoms at a younger age in the relapsing group. No significant sex differences were observed in disease history parameters, including median time between first symptoms and diagnosis, median disease duration, relapses within 2 years prior to study entry, or treatment at study entry. Men had a non-significant tendency toward higher EDSS scores at study entry in the relapsing group. After adjustment for age, disease duration, treatment, recent inflammatory activity, and BMI, this tendency remained. In progressive MS, no significant sex differences in EDSS were observed in either unadjusted or adjusted analyses.

The female-to-male ratio of approximately 2.1:1 in the relapsing type was consistent with other studies (Rommer et al., 2020). In PPMS, the sex distribution approached parity, with a female-to-male ratio of 0.73:1 in PPMS (36 women, 49 men) indicating a near-equal distribution in line with published reports (Westerlind et al., 2016). SPMS, which develops from RRMS (Ziemssen et al., 2020) with symptoms gradually and steadily worsening over time leading to increased disability (Ziemssen et al., 2020), demonstrates a female predominance (Bashir and Whitaker, 1999) as shown in our study (female-to-male ratio of 1.34:1). We hypothesize that this could be due to the female preponderance in RRMS, from which SPMS develops. Nevertheless, caution is warranted in interpreting these results given the limited sample sizes in both the SPMS and PPMS.

Concerning BMI, our study found women having lower BMIs than men in relapsing MS and SPMS. Interestingly, this pattern was not observed in PPMS, despite a similar sample size to the SPMS sample. Data from the Swiss statistical office has demonstrated a similar trend in the general population, suggesting that the lower BMI in women could be due to a geographical context rather than MS related mechanisms (Federal Statistical Office, 2017). Nevertheless, recent studies have shown interesting trends concerning BMI sex differences in person with MS (PwMS). Thévoz et al. (2024) showed that in both women and men, PwMS had an increase in central obesity compared to the control group (while having the same BMI). Given that central obesity contributes to metabolic syndrome, which is associated with systemic inflammation and potentially poorer MS disease evolution, addressing central obesity could represent a valuable intervention target (Thévoz et al., 2024). Furthermore, a study by Bove et al. (2016) demonstrated sex-specific associations between BMI and MS disability, with higher BMI correlating with greater disease severity in women but reduced severity in men. Although exploring the association between BMI and disease severity was beyond the scope of our study, we hypothesize that the lower BMI observed in women in this cohort may have implications for disease outcomes given these sex-specific effects. If the associations reported by Bove et al. (2016) apply to our population, the lower BMI in women could potentially be protective against disease progression. However, as our study design is cross-sectional and does not assess disease trajectories, we cannot determine whether the observed BMI differences are associated with differential disease outcomes. Longitudinal studies examining whether these BMI differences contribute to sex-specific disease trajectories would be valuable.

Age at first symptoms differed significantly between men and women in the relapsing type, with women experiencing symptoms at a younger age. Another cohort (Rommer et al., 2020) found a sex difference in relapsing type, with women also being younger than men. Men with PPMS had their first symptoms at a younger age compared to women. Thus, contradicting a previous study which showed no sex difference (Rommer et al., 2020) and suggested that sex may play a less pronounced role in the timing of symptom onset in progressive forms of MS. The reasons for this discrepancy remain unclear and may include cohort-specific factors, population differences, or methodological variations in symptom assessment and diagnosis.

No differences in MS family history were observed. This contrasts with documented sex-related genetic patterns in MS heritability, including the preferential maternal transmission of HLA-DRB1*15, higher carriage rates of HLA-DRB1 risk alleles in women, and increased transmission disequilibrium among female relatives (Bove and Chitnis, 2014). The absence of sex-specific familial clustering in this study may suggest that the presence of family history alone may not capture the nuanced sex-dependent genetic transmission patterns identified in molecular studies. In the SMSC approximately 13% of participants reported a family history of MS, a finding consistent with a systematic review (Harirchian et al., 2018).

The median time between first symptoms and diagnosis was not significantly different between men and women. A Danish study by Magyari and Koch-Henriksen (2022), involving 9,647 patients (3,028 men, 6,619 women), found that diagnostic delay did not differ significantly between sexes. Sex differences were observed in median disease duration at study entry. However, due to the open cohort design with variable enrollment times and the cross-sectional design, this measure is difficult to interpret as it reflects time from diagnosis to study entry rather than true disease course.

No significant sex difference concerning the median number of relapses 2 years prior to study entry were found. This finding is not consistent with the literature that demonstrated that women have higher relapse rates than men under the age of 50 in relapsing types (Kalincik et al., 2013; Magyari and Koch-Henriksen, 2022). Methodological differences could explain these discrepancies: our study captured relapses retrospectively over a 2-years window prior to study entry, whereas previous studies (Kalincik et al., 2013; Magyari and Koch-Henriksen, 2022) prospectively recorded relapses during longitudinal follow-up at regular clinical visits. Retrospective recall of relapses may underestimate true relapse rates and reduce sensitivity to detect sex-based differences compared to prospective registry-based monitoring. Women having higher relapse rates until the age 50, suggests hormonal influences on disease progression and relapse rate (Magyari and Koch-Henriksen, 2022). Women are predisposed to higher rates of inflammatory relapses and less disability progression than men (Gilli et al., 2020), possibly linked to hormonal influences, as pregnancy dramatically reduces relapse rates during the third trimester but increases them postpartum (Voskuhl and Gold, 2012). Notably the relatively high relapse burden observed in this cohort is consistent with the study’s inclusion criteria, which selected for patients initiating or switching to DMT, indicating more active disease at baseline.

Sex was not significantly associated with treatment usage in this cohort. Most treatments in this cohort were DMTs which are designed to alter the natural course of MS (Vosoughi and Freedman, 2010). A systematic review of 14 studies with 11,425 participants found no clear sex-based differences in DMT responses, though occasional sex-specific differences were observed for some clinical outcomes (Li et al., 2017). However, a recent study has shown that women are significantly underexposed to DMTs and highly effective DMTs compared to men (Gavoille et al., 2025). This seems to be linked to pregnancy-related considerations and associated therapeutic inertia whereby clinicians are reluctant to prescribe highly effective treatments to women of childbearing age, even when pregnancy is not imminent (Gavoille et al., 2025). Historically, women with MS were advised to discontinue DMTs before conception due to limited safety data (Lu et al., 2012), a practice that may contribute to ongoing patterns of undertreatment despite evolving evidence on the safety of certain medications during pregnancy (Iyer and Dobson, 2023). However, this cohort has the specificity to include persons who were about to initiate or change DMTs. This could have selected persons that were selected for treatment escalation and thus that have benefited from an appropriate treatment without delay.

When comparing disease severity, no sex differences were found concerning EDSS scores at baseline. In the relapsing type, EDSS scores showed a trend toward sex differences that approached statistical significance, whereas the progressive types showed no such pattern. In relapsing MS, while unadjusted and age-adjusted models showed no significant sex-related differences in EDSS, full adjustment for confounders revealed a borderline trend toward lower EDSS in women. This observed association is consistent with other studies showing that men generally experience worse clinical outcomes than women, with faster disability accumulation and more severe disease progression in relapsing MS (Magyari and Koch-Henriksen, 2022). This may reflect hormone-driven inflammatory activity in younger women leading to more relapses, versus a higher rate of underlying neurodegeneration in men leading to faster disability accumulation thus to higher disability scores (Magyari and Koch-Henriksen, 2022). In PPMS, defined by early and prominent neurodegeneration (Miller and Leary, 2007), disability accumulation rates were similar between women and men mirroring the literature (Ribbons et al., 2015). This observation is in line with the hypothesis that sex is primarily a prognostic factor in the inflammatory, relapse-onset phase of MS, but not in the progressive degenerative phase (Ribbons et al., 2015). In SPMS, baseline EDSS scores were similar between men and women consistent with the literature (Tremlett et al., 2008). However, it is critical to emphasize that our cross-sectional design captures only a snapshot at study entry and cannot assess disability accumulation or progression over time. The observed EDSS differences reflect baseline disability status, not trajectories.

### Limitations

Several limitations should be noted. First, the cross-sectional design captures only baseline characteristics at study entry and precludes assessment of disease trajectories, disability progression rates, or temporal relationships between variables. All findings represent associations at a single time point and cannot establish causality or predict future outcomes. Potential selection bias exists, as the cohort included participants who were either untreated, required a change in DMTs, initiated a new DMT, or were currently receiving treatment with a second or third generation DMT which may not fully represent the broader MS population thus limiting generalizability. This may introduce a potential bias, as the study population received highly effective treatment that may obscure certain sex-based differences. Third, while we conducted multivariable analyses to adjust for key confounders, residual confounding cannot be excluded, and our ability to establish independent effects of sex is limited by the observational design. Finally, data was limited to binary sex classification, which excluded consideration of gender diversity and more complex psychosocial gender aspects that may influence disease experience and outcomes.

### Strengths

Despite these limitations, this study has several notable strengths. First, it provides a comprehensive assessment of sex differences within the Swiss MS population, addressing an important gap in sex-specific research in this geographic context. Second, it offers a detailed descriptive analysis of MS characteristics in Switzerland, contributing valuable epidemiological data. Third, the study leverages the unique characteristics of the SMSC, which provides a well-characterized and homogeneous cohort with rigorous standardization protocols. The cohort’s focus on patients undergoing DMT switches provides insights into sex-related differences in a clinically relevant population at a critical therapeutic juncture, representing patients with active disease undergoing treatment optimization. Fourtly, the inclusion of both relapsing and progressive MS enables comparative analyses that highlight distinct clinical patterns between disease courses. Finally, the inclusion of both relapsing and progressive MS types enables comparative analyses that highlight distinct pathophysiological and clinical patterns between disease courses.

## Conclusion

In conclusion, this study examining sex differences in MS related to sociodemographic and clinical characteristics, disease history, and severity, found few differences beyond the female predominance in relapsing type and lower BMI in women. Multivariable analyses revealed a trend toward lower EDSS in women with relapsing MS after adjusting for confounders, though this did not reach statistical significance. No sex-related differences in EDSS were observed in progressive MS types. While investigating sex differences remains important for understanding disease heterogeneity, the risks of relying solely on binary female/male categories must be recognized. This approach can create perceptual biases that magnify perceived differences while masking substantial variation within groups and may lead to false positive claims of sex differences (Pape et al., 2024). Future research should move beyond binary sex categories to investigate the underlying mechanisms that drive disease variation. Further studies in Switzerland are needed, with emphasis on more nuanced measurements, prospective assessment of disease evolution, and examination of the specific biological and social factors that contribute to MS heterogeneity, ultimately informing the development of equitable and personalized interventions.

## Data Availability

The data analyzed in this study is subject to the following licenses/restrictions: Data access is restricted to protect participant privacy and requires approval from the Swiss Multiple Sclerosis Cohort (SMSC) Steering Committee. Data requests can be directed to the SMSC through institutional agreement. Requests to access these datasets should be directed to JK, jens.kuhle@usb.ch.

## Footnotes

1
http://www.neurostatus.net/
