# Supplementary material for: Sex differences in the development, treatment, and prognosis of multiple sclerosis in Switzerland
Source: Front Neurosci. 2026 Apr 8;20:1745599. doi: 10.3389/fnins.2026.1745599 (PMC13100965; doi:10.3389/fnins.2026.1745599)
Supplement: Supplementary file 1 [file Table_1.docx]

**Supplementary Material:**

Table S1. NFL z-score by sex for Relapsing MS

| **Relapsing MS (RRMS/CIS) (n = 1367)** | n | Men | Women | p-value | Overall |
| --- | --- | --- | --- | --- | --- |
| **n** |  | 440 | 927 |  |  |
| **Median NfL Z score with relapse in last 3 months (IQR)** | 264 | 1.7 (0.6, 2.8) | 1.6 (0.4, 2.6) | 0.2 | 1.7 (0.5, 2.6) |
| Missing, n % | 1,103 (81) | 356 (81) | 747 (81) |  |  |

Statistics (medians, interquartile ranges, percentages) and p-values were calculated using available data for each variable, excluding missing values.

Table S2. NFL z-score by sex for PPMS

| **PPMS (n = 85)** | n | Men | Women | p-value | Overall |
| --- | --- | --- | --- | --- | --- |
| **n** |  | 49 | 36 |  |  |
| **Median NfL z-score without relapse in last 3 months (IQR)** | 84 | 1 (0, 1.5) | 0.6 (-0.2, 1.4) | 0.354 | 0.8 (-0.1, 1.5) |
| Missing, n % | 1 (1.2) | 1 (2) | 0 (0) |  |  |

Statistics (medians, interquartile ranges, percentages) and p-values were calculated using available data for each variable, excluding missing values.

Table S3. NFL z-score by sex for SPMS

| **SPMS** (n=89) | n | Men | Women | p-value | Overall |
| --- | --- | --- | --- | --- | --- |
| **n** |  | 38 | 51 |  |  |
| **Median NfL z-score without relapse in last 3 months (IQR)** | 85 | 0,6 (−0,1–1,7) | 1 (0,1–1,9) | 0,438 |  |
| Missing, n % | 4 (4.5) | 3 (4.5) | 1 (2.6) |  |  |

Statistics (medians, interquartile ranges, percentages) and p-values were calculated using available data for each variable, excluding missing values.

Table S4. Ethnicity distribution by sex

| **Characteristic** | **Total***^1^* | **Men** | **Women** | **p-value** |
| --- | --- | --- | --- | --- |
| **Ethnicity** |  |  |  | 0.4 |
| Caucasian, n % | 1,513 (98.2) | 520 (98.7) | 993 (97.9) |  |
| Non-Caucasian, n % | 28 (1.8) | 7 (1.3) | 21 (2.1) |  |

Data presented as n (%). P-values calculated using Pearson's chi-squared test. MS: multiple sclerosis.

Table S5: Disease duration at study entry by sex (all MS types)

| **Characteristics** | **n** | **Median Disease Duration years, (SD)** | **p-value** |
| --- | --- | --- | --- |
| Men | 521 | 6.8 (1.7 - 14.1) | 0.8 |
| Women | 1009 | 6.9 (2.1 - 14.0) |  |
| Missing | 11 |  |  |

Data presented as median with interquartile ranges (IQR). P-value calculated using Mann-Whitney U test.

Table S6: Sex distribution by MS type at study entry

| **Characteristics** | **Total** | **Progressive** | **Relapsing** | **p-value** |
| --- | --- | --- | --- | --- |
| **Sex** |  |  |  | <0.001 |
| Men, n % | 527 (34.2) | 87 (50.0) | 440 (32.2) |  |
| Women, n % | 1,014 (65.8) | 87 (50.0) | 927 (67.8) |  |

Data presented as n (%). P-values calculated using Pearson's chi-squared test.

Table S7: Sex distribution by MS disease course at study entry

| **Disease course at study entry** | **Total (n)** | **Men, n%** | **Women, n %** | **Ratio F:M** | **p-value within each disease course** |
| --- | --- | --- | --- | --- | --- |
| CIS | 76 | 31 (40.8) | 45 (59.2) | 1.45 | 0.108 |
| PPMS | 85 | 49 (57.6) | 36 (42.4) | 0.73 | 0.159 |
| RRMS | 1291 | 409 (31.7) | 882 (68.3) | 2.16 | <0.001 |
| SPMS | 89 | 38 (42.7) | 51 (57.3) | 1.34 | 0.168 |

Data presented as n (%). P-values calculated using Pearson's chi-squared test. F:M: female-to-male ratio.

Table S8: Percentage of missing data by sex and MS subtype

| **Variable** | **Progressive – Men (%)** | **Progressive – Women (%)** | **Relapsing – Men (%)** | **Relapsing – Women (%)** |
| --- | --- | --- | --- | --- |
| Age at baseline | 0.0 | 0.0 | 0.0 | 0.0 |
| Age at first symptoms | 1.1 | 1.1 | 0.7 | 0.2 |
| BMI | 17.2 | 18.4 | 9.1 | 8.8 |
| Disease duration at baseline | 2.3 | 1.1 | 0.9 | 0.4 |
| EDSS at baseline | 1.1 | 0.0 | 0.2 | 0.6 |
| Ethnicity (Caucasian) | 0.0 | 0.0 | 0.0 | 0.0 |
| MS family history | 0.0 | 0.0 | 0.0 | 0.0 |
| NfL z-score (no recent relapse) | 2.3 | 3.4 | 22.0 | 23.7 |
| NfL z-score (with recent relapse) | 98.9 | 97.7 | 80.9 | 80.6 |
| Number of relapses (2 years before baseline) | 0.0 | 0.0 | 0.0 | 0.0 |
| Time from symptoms to diagnosis | 2.3 | 1.1 | 5.0 | 2.7 |
| Treatment category | 0.0 | 0.0 | 0.0 | 0.0 |

Table S9: Comparison of baseline characteristics between participants with and without available data for key variables

| **Baseline characteristic** | **BMI** | **EDSS** | **NfL with relapse** | **NfL without relapse** | **Disease duration** |
| --- | --- | --- | --- | --- | --- |
| Age at baseline | 0.8658 | 0.8013 | <0.001* | <0.001* | 0.5496 |
| Sex | 0.6960 | 0.7230 | 0.4097 | 0.1607 | 0.2675 |
| MS type | <0.001* | 1.000 | <0.001* | <0.001* | 0.1183 |
| EDSS at baseline | 0.6266 | NA | <0.001* | <0.001* | 0.8390 |
| Disease duration at baseline | 0.6954 | 0.8498 | <0.001* | <0.001* | NA |
| BMI | NA | 0.4518 | 0.8171 | 0.7900 | 0.7623 |
| Treatment category | 0.2679 | 0.2529 | <0.001* | <0.001* | 0.5792 |

Values represent *p*-values from statistical tests comparing participants with available versus missing data for each variable.
* p < 0.05.
NA: not applicable.
